# Expression of hepcidin mRNA is uniformly suppressed in hepatocellular carcinoma

**DOI:** 10.1186/1471-2407-8-167

**Published:** 2008-06-09

**Authors:** Hiroaki Kijima, Tokihiko Sawada, Naohisa Tomosugi, Keiichi Kubota

**Affiliations:** 1 Second Department of Surgery, Dokkyo University School of Medicine, Kitakobayashi 880, Mibu, Shimotsuga, Tochigi 321-0293, Japan; 2 Proteomics Research Unit, Division of Advanced Medicine, Medical Research Institute, Kanazawa Medical College, Daigaku 1-1, Uchinada, Kanazawa 920-0293, Japan

## Abstract

**Background:**

The present study evaluated the expression of hepcidin mRNA in hepatocellular carcinoma (HCC).

**Methods:**

Samples of cancerous and non-cancerous liver tissue were taken from 40 patients with HCC who underwent hepatectomy. Expression of hepcidin mRNA was evaluated by real-time PCR, and compared in tumors differing in their degree of differentiation, number of tumors, and vessel invasion. Correlations between hepcidin expression and the interval until HCC recurrence, and the serum concentration of hepcidin were evaluated, together with the expression of mRNAs for other iron metabolism molecules, ferroportin and transferrin receptor 2 (Trf2).

**Results:**

Hepcidin mRNA expression in non-cancerous and cancerous tissues was 1891.8 (32.3–23187.4) and 53.4 (1.9–3185.8), respectively (*P *< 0.0001). There were no significant differences in hepcidin expression among tumors differing in their degree of differentiation, number of tumors, or vessel invasion. There was no significant correlation between hepcidin expression and the interval until HCC recurrence. The serum concentration of hepcidin-25 was not correlated with hepcidin-mRNA expression. Finally, there were no significant differences in the expression of mRNA for ferroportin and Trf2 between cancerous and non-cancerous tissues.

**Conclusion:**

Expression of hepcidin mRNA is strikingly suppressed in cancerous, but not in non-cancerous tissues, in patients with HCC, irrespective of ferroportin or Trf2 expression. Uniform suppression of hepcidin may be linked to the development of HCC.

## Background

Hepatocellular carcinoma (HCC) is a major cause of death worldwide [1], and chronic inflammatory stress caused by hepatitis viruses B and C plays a major role in HCC carcinogenesis [2]. Furthermore, some studies have indicated that iron overload is a major risk factor for development of HCC [3]. Iron overload leads to the generation of reactive oxygen species (ROS), which cause chronic inflammation in the liver [4]. Iron accumulation is associated not only with the genetic iron overload disorder, hemochromatosis, but also with acquired hemosiderosis after chronic viral hepatitis or in fatty liver [5-7].

Hepcidin is a key molecule for maintenance of iron homeostasis [8]. Hepcidin is produced in hepatocytes [9], and binds to, internalizes, and degrades ferroportin-1 [10], resulting in a decrease of serum iron concentration and an increased intracellular iron content [11]. There is a considerable body of evidence that expression of hepcidin is altered in various types of diseases. Anemia of inflammation induces overexpression of hepcidin [12,13]. However, no studies have investigated the expression of hepcidin in HCC.

In this study, we investigated the expression of hepcidin in HCC and showed, for the first time, that it is strikingly suppressed in this cancer.

## Methods

### Patients

Forty patients who had undergone hepatic resection and had been diagnosed as having HCC by histological examination were included in the present study. The documented consent was obtained from the each patient. The patients' background factors are summarized in Table 1. The mean age was 62.1 ± 11.3 years, and there were 29 males and 11 females. The number of patients positive for HCV, HBV, and both HCV and HBV was 11, 8, and 12, respectively. Liver cirrhosis was observed in 22 patients and chronic hepatitis was diagnosed in 15; only 3 patients lacked chronic hepatitis or liver cirrhosis.

**Table 1:** 

Patients	n = 40
Age (year)	62.1 ± 11.3
Sex	
Male	n = 29
Female	n = 11
Virus	
HCV+	n = 11
HBV+	n = 8
HCV+HBV+	n = 12
HCV-HBV-	n = 9
Cirrhosis	
Yes	n = 22
No normal	n = 3
CH	n = 15
Tumor differentiation	
Well	n = 4
Moderately	n = 32
Poorly	n = 4
Number of tumors	
1	n = 29
2	n = 5
3	n = 4
4-	n = 2
Vessel invasion	
Negative	n = 31
Positive	n = 9

### Real-time PCR

For real-time PCR, samples of both non-cancerous and cancerous liver tissue were available for all 40 patients. Surgical samples weighing 500 mg were stored in liquid nitrogen immediately after the operation, and kept at -80°C until RNA extraction. Total RNA from each sample was isolated using a Total RNA Isolation Kit (Macherey-Nagel, Düren, Germany). Reverse transcription reactions were performed using a Rever Tra Ace α-First Strand cDNA Synthesis Kit (Toyobo, Osaka, Japan). Briefly, 1 μg of total RNA, oligo dT-primer, and dNTPs were incubated at 65°C for 5 min, then 10 μL of a cDNA synthesis mixture was added and the mixture was incubated at 50°C for 50 min. The reaction was terminated by adding 1 μL of RNaseH and incubating the mixture at 37°C for 20 min.

Real-time PCR was performed with an ABI Prism 7700 sequence detector (Applied Biosystems, Warrington, UK). The PCR reaction was carried out in a final volume of 2 μL cDNA, 12.5 μL 2 × SYBR Green (Applied Biosystems), 0.5 μL of 25 nM sense and antisense primers, and H_2_O up to 25 μL. The PCR conditions consisted of 40 cycles at 95°C for 15 s and 60°C for 60 s. The sequences of the primers were as follows: GAPDH: sense-primer 5'-CCACCCAGAAGACTGTGGAT-3', anti-sense 5'-TTCAGCTCAGGGATGACCTT-3' ; hepcidin: sense-primer 5'-CACAACAGACGGGACAACTT-3', anti-sense 5'-CGCAGCAGAAAATGCAGATG-3' [14]; ferroportin-1: sense-primer 5'-CGAGATGGATGGGTCTCCTA-3', anti-sense 5'-ACCACATTTTCGACGTAGCC-3' ; transferrin receptor-2 (Trf2): sense-primer 5'-CCTAGGCTCCCCTTATCACC-3', anti-sense 5'-TCACCATGGAGGAAAAGGTC-3'.

The level of expression was calculated using the formula: Relative expression (t) = (Copy number of target molecule/Copy number of GAPDH) × 1000 [14]. Samples were assayed in triplicate. Means and standard deviations were calculated from the data obtained. For each sample, at least three assays were performed. The *t *value was calculated from the mean of three different assays.

### Disease-free survival and expression of hepcidin mRNA

For analysis of the correlation between hepcidin mRNA expression and disease-free patient survival, 15 of the 40 patients who developed HCC recurrence within the study period were included. As only 3 patients died of HCC in the observation period, overall survival analysis was not performed.

### Measurement of serum hepcidin-25, iron, ferritin, and total iron binding capacity (TIBC)

Serum hepcidin-25, iron, ferritin, and TIBC were measured in blood samples collected from 15 patients with HCC. Serum hepcidin-25 concentration was measured using LC-MS/MS at Medical Care Proteomics Biotechnology Co., Ltd. (Kanazawa, Japan). The measurement of serum hepcidin-25 has been described elsewhere [15]. The normal serum hepcidin-25 level was 22.2 ± 12.3 ng/mL. Analyses of the correlation between serum hepcidin concentration and hepcidin mRNA expression were performed using the serum samples and surgical specimens from these 15 patients. Serum concentrations of iron, ferritin, and TIBC were measured at BML, Inc. (Tokyo, Japan). The normal serum levels of iron, ferritin, and TIBC were determined according to the data from BML, Inc. The normal serum iron values for men and women were set at 55–190 μg/dL and 45–145 μg/dL, respectively. The normal serum ferritin values for men and women were set at 20–250 ng/mL and 5–120 ng/mL, respectively. The normal TIBC values for men and women were set at 250–380 μg/dL and 250–450 μg/dL, respectively.

### Statistical analyses

Comparisons between two groups were analyzed by Mann-Whitney test (two-sided). One-factor ANOVA was used for comparisons between more than 3 groups. Correlations were analyzed using Spearman's correlation coefficient by rank test. A probability value of *P *< 0.05 was considered to indicate statistical significance.

## Results

### Hepcidin mRNA expression is suppressed in hepatocellular carcinoma

**Figure 1:**
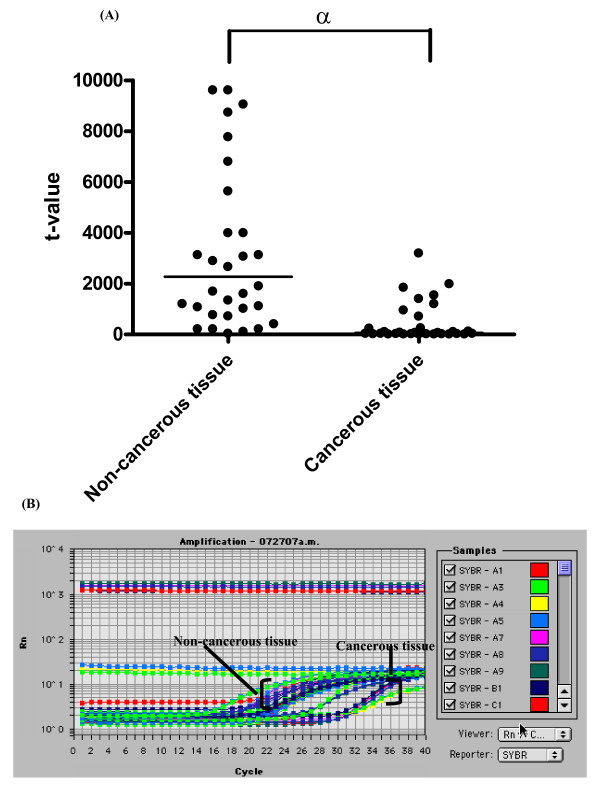
**Suppression of hepcidin mRNA in cancerous tissue from patients with HCC**. (A) The median t values (horizontal bar) for hepcidin mRNA (n = 40) in non-cancerous and cancerous tissue were 1891.8 (32.3–23187.4) and 53.4 (1.9–3185.8), respectively (*P *< 0.05). α: statistically significant. (B) Representative results of real-time PCR of non-cancerous and cancerous tissue are shown.

The median t values for hepcidin mRNA in non-cancerous and cancerous tissues were 1891.8 (32.3–23187.4) and 53.4 (1.9–3185.8), respectively (*P *< 0.0001) (Fig. 1). Expression of hepcidin mRNA was significantly inhibited in cancerous tissue.

**Figure 2:**
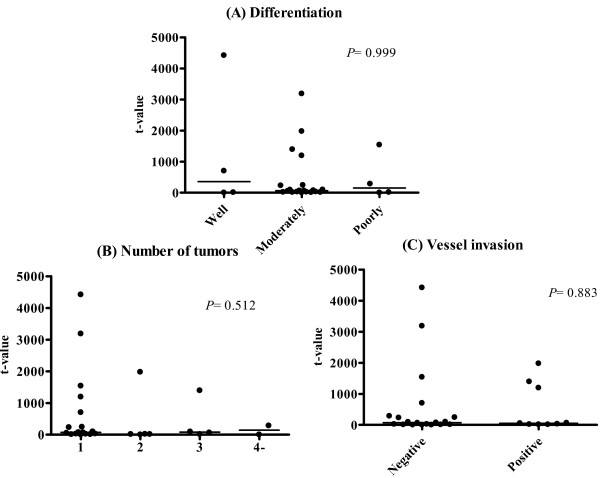
**Hepcidin mRNA expression in tumors differing in their degree of differentiation, number of tumors, and vessel invasion**. Hepcidin mRNA expression did not differ among (A) well (n = 4), moderately (n = 32), and poorly (n = 4) differentiated carcinoma, (B) patients with 1 (n = 29), 2 (n = 5), 3 (n = 4), and 4 or more (n = 2) HCCs, and (C) negative (n = 31) and positive (n = 9) for vessel invasion.

Figure 2 shows the median t values for hepcidin mRNA in cancerous tissues varying in their degree of tumor differentiation, number of tumors, and vessel invasion. Median t values for hepcidin mRNA in well, moderately and poorly differentiated HCC were 355.9, 64.9, and 150.4, respectively (*P *= 0.999). Median t values for hepcidin mRNA in patients who had 1, 2, 3 and 4 or more HCCs were 71.5, 13.8, 79.2, and 144.0, respectively (*P *= 0.512). Median t values for hepcidin mRNA in patients who were negative and positive for vessel invasion were 68.8 and 53.4, respectively (*P *= 0.883).

**Figure 3:**
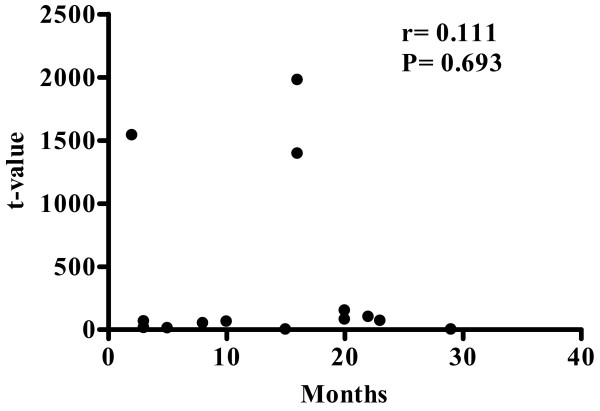
**Correlation between hepcidin mRNA expression and period until HCC recurrence**. Hepcidin mRNA expression (n = 15) was not correlated with the period until. HCC recurrence (r = 0.111, *P *= 0.693).

Next, we investigated the correlation between the expression of hepcidin mRNA and patient disease-free survival period (Fig. 3). There was no significant correlation between the expression of hepcidin mRNA and disease-free survival period (Fig. 3, r = 0.111, *P *= 0.693).

**Figure 4:**
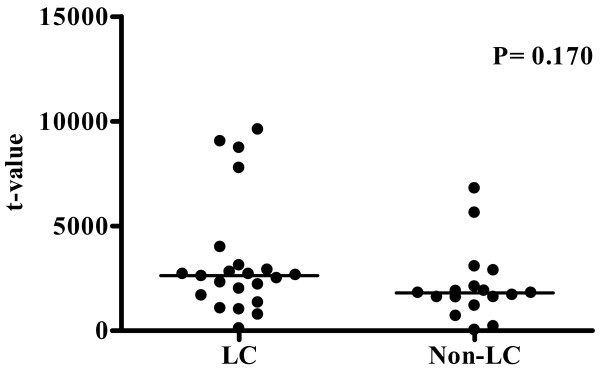
**Hepcidin mRNA expression in cirrhotic and non-cirrhotic liver tissue**. Hepcidin mRNA expression in non-cancerous liver tissue did not differ between HCC patients with (LC, n = 22) and without (Non-LC, n = 18) cirrhosis.

Figure 4 shows the expression of hepcidin mRNA in non-cancerous tissue in patients with and without cirrhosis. There was no significant difference in the expression of hepcidin mRNA expression between the two groups (*P *= 0.170).

### Ferroportin-1- and Trf2 mRNA expression is not suppressed in hepatocellular carcinoma

**Figure 5:**
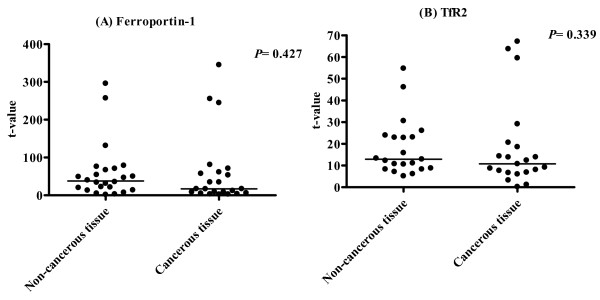
**Expression of mRNA for ferroportin-1 and Trf in HCC**. Expression of mRNA for ferroportin-1 (A) and Trf (B) did not differ between cancerous and non-cancerous tissue in patients with HCC.

We also investigated the expression of mRNA for two other molecules, ferroportin-1, and Trf2, which play a crucial role in iron homeostasis (Fig. 5). Expression of ferroportin mRNA in non-cancerous and cancerous tissue was 37.9 (1.9–295.9) and 17.2 (2.6 – 794.9), respectively (P = 0.427), whereas that of Trf2-mRNA was 12.9 (5.2 – 54.8) and 10.8 (0.2 – 67.2), respectively (P = 0.339).

### Hepcidin mRNA expression is not correlated with serum hepcidin-25 concentration

**Figure 6:**
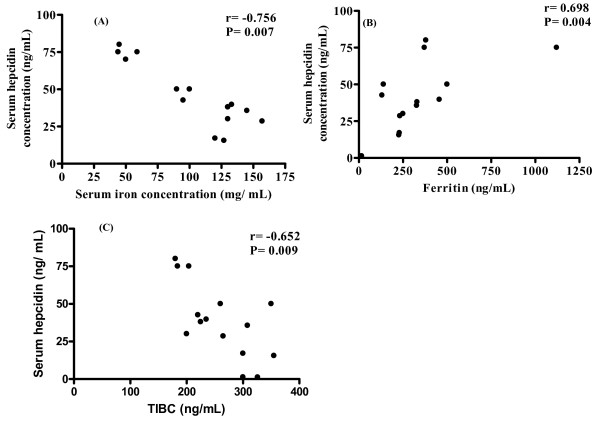
**Correlations between serum concentration of hepcidin and iron, ferritin, and TIBC**. Serum concentration of hepcidin (n = 15) was significantly correlated with serum concentration of iron (A), ferritin (B), and TIBC (C).

We then investigated the serum concentration of biochemical markers of iron metabolism. Among 15 patients with HCC, the serum iron level was low in only 2 (52.3 ± 2.6 mg/mL), and normal in the other 13 (131.4 ± 23.4 mg/dL). The serum ferritin level was high in 4 patients (414.4 (328.2–1121.0) ng/mL) and normal in 11 (179.5 (14.0–232.9) ng/mL). TIBC was low in 4 patients (194.0 ± 14.1 ng/mL) and normal in 11 (284.8 ± 28.3 ng/mL). The serum hepcidin-25 value was high in 5 patients (42.6 ng/mL (35.6–75.0)) and normal in 10 (15.5 ng/mL (1.2–28.5)). There were significant correlations between the serum levels of hepcidin-25 and iron (Fig. 6A, r = -0.756, *P *= 0.007), hepcidin-25 and ferritin (Fig. 6B, r = 0.698, *P *= 0.004), and hepcidin and TIBC (Fig. 6C, r = -0.652 *P *= 0.009).

**Figure 7:**
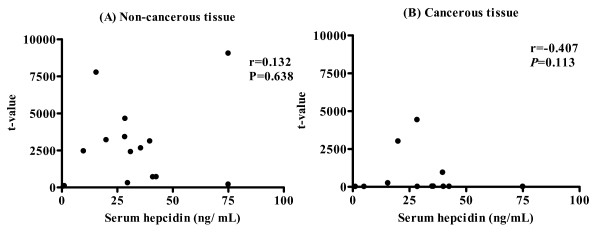
**Correlation between expression of hepcidin mRNA and serum concentration of hepcidin**. Expression of hepcidin mRNA was not correlated with serum concentration of hepcidin (n = 15) in non-cancerous or cancerous tissues.

The serum hepcidin-25 concentration was not significantly correlated with expression of hepcidin mRNA in non-cancerous tissue (Fig. 7A, r = 0.132, *P *= 0.638) or cancerous tissue (Fig. 7B, r = -0.407, *P *= 0.248).

## Discussion

Hepcidin is a molecule playing a key role in iron homeostasis. It is produced by the liver, and inhibits intestinal iron absorption by enterocytes in the duodenum [16] and also release of iron by macrophages and hepatocytes [17].

Production of hepcidin is controlled by various stimuli and factors. Production of hepcidin is stimulated by iron overload, inflammation, and proinflammatory cytokines such as IL-6, whereas it is decreased by iron deficiency and erythropoiesis, leading to iron accumulation in the body [13].

It is well known that HCC develops in more than 40% of patients with hemochromatosis [18]. On the other hand, iron is an essential nutrient for cell growth, and cancer cells in particular require iron in order to proliferate [19]. The present study clearly demonstrated that expression of hepcidin mRNA was suppressed universally in HCC, irrespective of the degree of tumor differentiation, and was not correlated with the period until cancer recurrence. Expression of hepcidin was maintained in non-cancerous liver tissue of patients with HCC, and the level of hepcidin expression did not differ between cirrhotic and non-cirrhotic liver (Figure 4). Although the mechanism reponsible for suppression of hepcidin mRNA expression in HCC remains unclear, suppression of hepcidin transcription contradicts the previously proposed scheme for iron homeostasis in cancer cells, because cancer cells must retain iron in order to proliferate. However, suppression of hepcidin is rational because duodenal enterocytes transfer iron to plasma, resulting in an increase of total body iron content.

Recently, Weizer-Stern et al. reported that activation of the tumor suppressor gene p53 stimulates the expression of hepcidin [20]. The promoter region of the hepcidin gene (*HAMP*) contains a putative p53 response element. Inactivation or mutation of the p53 gene has been detected in various types of human cancer [21], including HCC [22]. Suppression of hepcidin expression may be linked to the altered expression and function of p53.

Ferroportin-1 is an iron transporter protein produced in hepatocytes as well as duodenal enterocytes, macrophages, and placental cells [23]. Ferroportin-1 exports iron from the intracellular to the extracellular space to increase the iron content of plasma, and its expression is regulated by intracellular iron content. Hepcidin binds to, internalizes, and degrades ferroportin-1, resulting in an increase of the intracellular iron content [24]. TfR2 is a transmembrane type II protein expressed in the liver by hepatocytes, and binds to transferrin [25]. It has been reported that hepcidin expression is suppressed in TfR2 knockout mice, suggesting that TfR2 gene expression is located upstream from hepcidin gene expression [26]. An increase of TfR2 results in an increase of hepcidin production. In the present study, expression of mRNA for ferroportin-1 and TfR2 did not differ between non-cancerous and cancerous tissues, whereas the expression of hepcidin was uniformly suppressed in cancerous tissues. The expression of hepcidin was suppressed in HCC regardless of the level of ferroportin-1 and TfR2 expression.

We found that serum hepcidin-25 concentration was correlated with the levels of serum iron and ferritin, but not with the level of hepcidin mRNA expression in either cancerous or non-cancerous liver tissue (Figure 7). Hepcidin is produced in patients with HCC, from non-cancerous liver tissue, even though production is inhibited in cancerous tissue.

## Conclusion

Expression of hepcidin mRNA is constitutively suppressed in cancerous, but not in non-cancerous liver tissue of patients with HCC. The precise mechanism responsible for the suppression of hepcidin in HCC should be investigated further, focusing on its role in the development and maintenance of this cancer.

## Competing interests

The authors declare that they have no competing interests.

## Authors' contributions

HK carried out whole process of the experiment. TS designed, planned, and instructed the experiment. TN carried out the measurement of the serum concentration of hepcidin and other molecules. KK obtained surgical samples and instructed the experiment.

## Pre-publication history

The pre-publication history for this paper can be accessed here:


